# Impact of the COVID-19 Pandemic on Patients Affected by Non-Communicable Diseases in Europe and in the USA

**DOI:** 10.3390/ijerph18136697

**Published:** 2021-06-22

**Authors:** Catherine Pécout, Emilie Pain, Michael Chekroun, Claire Champeix, Claudie Kulak, Rita Prieto, Joris van Vugt, Kim Gilchrist, Anne-Félice Lainé-Pellet

**Affiliations:** 1 Viatris, 75014 Paris, France; catherine.pecout@viatris.com (C.P.); annefelice.laine@viatris.com (A.-F.L.-P.); 2 Carenity, 75008 Paris, France; michael@carenity.com; 3 Eurocarers, 1040 Brussels, Belgium; cc@eurocarers.org; 4 La Compagnie des Aidants, 94200 Ivry-sur-Seine, France; ckulak@lacompagniedesaidants.org; 5 Viatris, 28027 Madrid, Spain; rita.prieto@viatris.com; 6 Viatris, 2909 LD Capelle a/d IJssel, The Netherlands; joris.vanvugt@viatris.com; 7 Viatris, Collegeville, PA 19426, USA; kim.gilchrist@viatris.com

**Keywords:** COVID-19, non-communicable diseases, patient experience, health care impact, isolation, stress, source of information, behavior, access

## Abstract

An international online patient community, Carenity, conducted a patient study in two independent waves among adults affected by non-communicable diseases (NCDs) in Europe and in the United States of America (USA). The study aimed to assess the real time impact of the coronavirus disease 2019 (COVID-19) on the medical conditions of patients with NCDs, their access to health care, and their adaptation to daily life as well as to describe their sources of information on COVID-19 and their needs for specific information and support. During the pandemic, 50% of the patients reported a worsening of their medical condition, and 17% developed a new disease. Additionally, 26% of the respondents reported an impact of the pandemic on regular/long-term treatment intake. 54% of the patients felt very or completely socially isolated and reported a strong impact of the COVID-19 pandemic on their stress level and state of mind, with higher levels observed in the USA compared to Europe. 59% of the respondents wished to have received additional information regarding the risks associated to their medical condition during the pandemic. Television was the most used source of information, whereas physicians were the most trusted one. This study describes the substantial impact of the COVID-19 pandemic on NCD patients.

## 1. Introduction

Non-communicable diseases (NCDs), also known as chronic diseases, result from genetic, physiological, environmental, and behavioral factors. These long-term and sometimes life-long pathologies include cardiovascular diseases, cancers, chronic respiratory diseases, diabetes as well as mental health and neurological disorders [1]. Since 2015, NCDs have surpassed infectious diseases as the leading cause of morbidity, disability, and death worldwide [2,3]; in 2019, they were accountable for 71% of all-caused deaths, the major global cause being cardiovascular diseases (17.9 million deaths), followed by cancer (9.0 million), respiratory diseases (3.8 million), and diabetes (1.6 million) [3]. Many of these premature deaths occurred before the age of 70, with most of them being preventable and treatable. While multiple interventions in diverse policy areas have been recommended to increase the prevention and control of NCDs and have achieved a noticeable reduction of their impact [1], their global economic burden remains high and increasing because of the growing age of the worldwide population [4,5]. Among all NCDs, mental illnesses and cardiovascular diseases account for the highest financial burden [6]. In particular, mental health pathologies, like major depressive disorders or anxiety, lead to a sedentary lifestyle, which in turn may increase the predisposition toward other disease conditions (comorbidities). NCDs and the associated impairments and comorbidities greatly impact a patient’s quality of life. The leading environmental and behavioral risk factors for NCDs are tobacco use, air pollution, physical inactivity, unhealthy diet, and the harmful use of alcohol; many individuals combine several risk factors associated with the development of NCDs [7,8]. In the management of NCDs, primary care physicians are often the first point of contact for patients [9]; relationships established between healthcare professionals (HCPs) and patients are primordial for health monitoring, providing necessary education on relevant pathologies and treatment adherence [3]. Informal carers/caregivers (a person who provides—usually—unpaid care to someone with a chronic illness, disability, or other long-lasting health or care need outside of a professional or formal framework) are also important in the management of NCDs as well as for the physical and mental well-being of the persons coping with NCDs.

In December 2019, a novel coronavirus infection caused by SARS-CoV-2 first emerged in China; following its worldwide spread, the World Health Organization (WHO) declared the coronavirus disease 2019 (COVID-19) as a pandemic on 11 March 2020. Between March and May to June 2020, governments of most countries worldwide declared health emergency states and imposed confinement restrictions (lockdowns) to tackle the pandemic and avoid an overflow of intensive care units in hospitals. With the rapid spread of COVID-19 across the world, broad and sudden disruptions to health services occurred, mostly due to restrictions in public transport hindering access to health care facilities for patients, insufficient Personal Protective Equipment (PPE), insufficient clinical staff because of redeployment to COVID-19 units as well as the unavailability of essential medicines and services [10]. Those disruptions in health care might have had long-term consequences for patients living with NCDs, especially those particularly vulnerable and requiring regular or long-term care. Additionally, patients with pre-existing NCDs have been declared to be more at risk of serious illness or death from coronavirus infection [10,11]. Due to the chronic nature of their pathology, NCD patients most often need access to essential health or rehabilitation services over long periods of time. In a WHO report based on a global survey, 59% of countries reported that access to outpatient services was restricted to some degree, with rehabilitation services being the most likely impacted (50% of countries reported partial disruption and 12% complete disruption). In Europe, 79% of countries reported disruptions to rehabilitation services [10,12]. As noted by Caminiti et al., admissions for NCDs in northern Italy dropped by approximately one third in 2020 compared to 2019 [13]. Similarly, Jones et al. described a substantial decrease in screening, case identification, and referral in symptomatic cancer diagnosis in the UK for the same period, with all UK national cancer screening programs being suspended [14]. Likewise, a substantial reduction in the number of patients with acute coronary syndrome admitted to hospital in England by the end of March 2020 was observed, which likely resulted in an increase of deaths and long-term complications of myocardial infarction [15]. Additionally, a study reported that 2.3 million cancer surgeries had already been cancelled or postponed globally during the first lockdown due to COVID-19; overall, the authors predicted that 28 million elective surgeries would be cancelled or postponed worldwide in 2020 [16]. In the USA, 92% of elective vascular surgeries were cancelled at the beginning of the pandemic in April 2020 [17].

The objective of this study, performed in two independent waves, was to understand how patients with NCDs—a population that is particularly vulnerable in the context of the pandemic and lockdown—have been affected during the COVID-19 outbreak in Europe and in the USA. To do so, this study aims to evaluate: (1) the impact of COVID-19 and lockdowns on different aspects of NCD patients’ lives (treatment compliance and access to medication, communication and consultation with HCPs, development of new behaviors, impact on work situation, impact on mental health), and (2) patients’ expectations towards information on COVID-19 (topics of concern, most consulted and trusted sources of information, needs for additional information and services).

## 2. Materials and Methods

### 2.1. Study Design

Carenity, an international online patient community, conducted a study based on an online survey among adult patients affected by NCDs. The study aimed to understand the impact of the COVID-19 pandemic on patients with NCDs in terms of the evolution of their medical condition and access to physicians and treatments, and to describe their sources of information regarding COVID-19 and their needs for specific information and support. This study was conducted in two waves: a first wave following the first strict lockdown and a second one some months later, while most countries were still under sanitary restrictions due to COVID-19 infection (strict or soft lockdown depending on the ongoing stage of the pandemic).

### 2.2. Study Population

The study population included registered Carenity members. Carenity is a leading digital patient platform with 400,000 patients and caregivers worldwide (88% patients/12% caregivers). Through Carenity communities, members can share experiences and find health-related information on their condition as well as contribute to medical research by participating in online studies to highlight their patient experience.

Eligible patients were defined as adult patients (18 years old or older) living in Belgium, France, Germany, Italy, Spain, the UK, or the USA, having at least self-reported a diagnosed NCD and who were members of the Carenity community. The NCDs included metabolic diseases, chronic respiratory diseases, mental health diseases, cardiovascular diseases, and cancers. Because of the small sample size of the respondents in Belgium (Table 1) and their belonging to the French speaking area, Belgian patients who answered the survey on the French Carenity platform were reported together with the French population for the purposes of the study.

Patients meeting the inclusion criteria completed an initial survey (wave 1) and/or a second survey (wave 2) to self-report the impact of the COVID-19 pandemic and lockdown on their care pathway. The two survey periods were enrolled independently, and patients who participated in wave 1 could self-select, but were not required, to complete the wave 2 survey. The study population was defined by all patients who completed the questionnaire (wave 1 and/or wave 2).

### 2.3. Ethical Considerations

This study was conducted in accordance with the Declaration of Helsinki and following the principles of good clinical practice. Prior to data collection, all patients gave their informed online consent (patients were informed that the treatment of their health data is conducted based on their explicit consent, formalized by the click of the “Start” button at the bottom of the survey’s information page, and clicking on the button), which allowed for data collection and publication. The participants’ privacy and confidentiality was guaranteed following the USA health care personal data protection law (HIPAA, Health Insurance Portability and Accountability Act) as well as European laws and regulations (GDPR, General Data Protection Regulation). Ethical review and approval were waived for this study, as the aim of the study is to determine the patients’ insights from their experiences, and informed consent was given by all patients prior to answering the survey.

### 2.4. Data Collection

Carenity has setup a barometer (the 2 independent waves) to measure the impact of COVID-19 on access to health care and quality of life for chronic patients in real time. Members of the Carenity NCDs communities were asked to complete the same online survey at two distinct periods. In the countries involved in this study, a strict lockdown was in place for several weeks between March and June 2021, depending on the country; from 22 June 2020 to 14 August, patients reported experience during this first lockdown in Europe and in the USA (data from wave 1). The data regarding the impact of the second lockdown were collected in real time (prospectively) from 2 November 2020 to 29 December 2020 (data from wave 2). During the second survey sample (wave 2), not all of the countries included in this study had a further strict lockdown at that point in time; however, all of the maintained strict sanitary restrictions concerning displacements and physical and social distancing were still in practice in those countries. During the second wave, many EU countries were considering a stricter lockdown, which began to be applied during the yearend holidays. The collected data of both waves were analyzed in two separated cohorts; patients could participate in both waves, but this was not mandatory. For comparison purposes, the same survey including 39 closed-ended questions was used for both waves. The questionnaire was validated to ensure compliance for each country involved as well as by native speakers, as it was programmed in local languages for the different Carenity worldwide platforms. The collected data included self-reported sociodemographic and medical profiles as well as information about the patients’ health-related quality of life (HR-QoL) during the COVID-19 pandemic and lockdown and their needs towards the pandemic. The following notions were especially evaluated: disability (disability is characterized by the limitations or reductions in the individual’s ability to participate in their activities of daily living due to a disease or health condition) [18], social isolation (social isolation is the inadequate quality and quantity of social relations with other people at the different levels where human interaction takes place (individual, group, community and the larger social environment)) [19], depressive symptoms (depressive symptoms can vary from mild to severe and can include: (1) feeling sad or having a depressed mood, (2) loss of interest or pleasure in activities once enjoyed, (3) changes in appetite—weight loss or gain unrelated to dieting, (4) trouble sleeping or sleeping too much, (5) loss of energy or increased fatigue, (6) increase in purposeless physical activity (e.g., the inability to sit still, pacing, handwringing) or slowed movements or speech (these actions must be severe enough to be observable by others), (7) feeling worthless or guilty, (8) difficulty thinking or concentrating or making decisions, and (9) thoughts of death or suicide [20]), and regular treatment/long term treatment (there is no commonly accepted definition of long-term treatment. In our survey, it has been defined as a treatment that was taken for at least three months. No differences have been made between regular and long-term treatment). Data collected on the Carenity platform are hosted in France on a secured computer server in accordance with the requirements of the “Commission Nationale de l’Informatique et des Libertés” (CNIL)—declaration number n°1484083, dated 29 March 2011.

### 2.5. Statistical Analysis

Descriptive univariate and multivariate analyses were performed using the statistical software R (version 3.5.2 (The R Foundation, Vienna, Austria)). For quantitative data, the following indicators were calculated: mean, range (minimum, maximum), frequency—*n* (%). Multivariate analysis included subgroup analyses by country. A multiple correspondence analysis was also performed using the statistical software R (version 3.5.2) for the clustering analysis, and internal validation measures were used to assess the quality of the clustering. The resulting clusters were thus analyzed through a hierarchical cluster analysis. The *p*-values were calculated with a confidence interval of 95%. A *p* value < 0.05 was considered statistically significant.

## 3. Results

### 3.1. Characteristics of the Study Population

**Figure 1:**
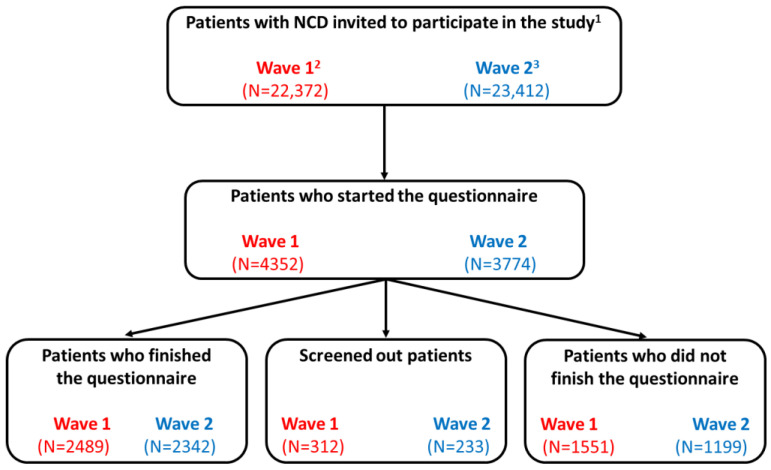
Study population flowchart of both study waves. ^1^ Patients who were members of the Carenity non-communicable disease (NCD) communities and had agreed to receive invitations to participate in questionnaires; ^2^ A first study wave was conducted between 22 June 2020 and 10 August 2020; ^3^ A second study wave was performed between 2 November 2020 and 29 December 2020.

Overall, 22,372 members of the Carenity NCDs communities were invited to participate in the study after the first lockdown (wave 1) from 22 June 2020 to 14 August 2020 and 23,412 after the second lockdown (wave 2) from 2 November 2020 to 29 December 2020 (Figure 1). Among the 4352 patients who started the questionnaire in the first wave (3774 in wave 2), the respondent population consisted of 2489 patients (2342 in wave 2) who filled it completely with valid answers; 195 patients answered both waves.

As few differences were observed between results from wave 1 and wave 2, the results of wave 1 are presented in the article, and only the differences found between both waves are highlighted for clarity.

#### 3.1.1. Sociodemographic Profile

In wave 1, a total of 2489 patients with NCDs living in six different countries answered the survey for the Carenity community. Table 1 summarizes the sociodemographic profile of patients. Most patients were women (*n* = 1602/2489, 64%) and the mean age was 53 years (range, 18–91), with 66% (*n* = 1649/2489) of the patients being under 60 years old. During the lockdown, 80% (*n* = 1957/2489) of respondents were living in Europe (25% (*n* = 615/2489) in France or Belgium, 18% (*n* = 458/2489) in the UK, 14% (*n* = 354/2489) in Spain, 13% (*n* = 313/2489) in Italy, 10% (*n* = 247/2489) in Germany, and 20% (*n* = 502/2489) in the USA. Overall, respondents were predominantly living in small cities (*n* = 637/2489, 26%), followed by mid-sized cities (*n* = 568/2489, 23%) or metropolitan cities (*n* = 542/2489, 22%). During the pandemic, most participants (*n* = 1418/2489, 56%) were residing in a house with a yard while 33% (*n* = 814/2489) lived in an apartment, 5% (*n* = 125/2489) in a house without a yard, and 3% (*n* = 63/2489) in a studio apartment. Including children, most households housed two persons (*n* = 944/2489, 38%) during the pandemic and 24% (*n* = 598/2489) of households included more than three persons. Sociodemographic profiles from both surveys showed a very good degree of consistency between the NCD population in both waves.

#### 3.1.2. Clinical Characteristics

Table 2 presents the clinical characteristics of the respondents in both waves. When asked about their chronic illness, 42% of participants of wave 1 (*n* = 1042/2489) answered metabolic diseases, 37% (*n* = 930/2489) respiratory diseases, 24% (*n* = 603/2489) cardiovascular diseases, and 23% (*n* = 577/2489) mental disorders. Among the most frequent conditions, patients mentioned type 2 diabetes (*n* = 832/2489, 33%), arterial hypertension (*n* = 575/2489, 23%), asthma (*n* = 562/2489, 23%), COPD (*n* = 459/2489, 18%), or depression (*n* = 412/2489, 17%). Compared to wave 1, a decrease in the number of cited respiratory diseases (*n* = 772/2342, 33% in wave 2 vs. *n* = 930/2489, 37% in wave 1) and an increased in the number of respondents with mental disorders (*n* = 597/2342, 25% in wave 2 vs. *n* = 577/2489, 23% in wave 1) was observed during wave 2. The noted increase in the proportion of mental disorders between the first and second lockdowns (from 23% to 30% of respondents) showed great disparity among the countries: an increase of 21% took place between the first and second lockdowns in the UK (*n* = 42/548, 9% vs. *n* = 104/346, 30% of mental disorders), 11% in France/Belgium (*n* = 56/615, 9% vs. *n* = 113/565, 20%), and 9% in Germany (*n* = 63/247, 26% vs. 127/363, 35%), whereas mostly no change was observed in the USA (*n* = 200/502, 40% vs. 157/412, 38%) or in Spain (*n* = 109/354, 31% vs. *n* = 108/374, 29%).

Overall, 45% (*n* = 1132/2489) of respondents considered that their chronic disease was disabling (disabling level of 6–10, 10 being the highest score = “very disabling”), with a mean disability level of 5.5 (range, 0–10). The second wave did not show any relevant difference compared to the first wave concerning this parameter.

In total, 90% (*n* = 2242/2489) of the respondents were currently taking regular/long-term treatments for their primary chronic illness.

Before the first lockdown caused by the COVID-19 pandemic, 41% (*n* = 1033/2489) of participants declared having experienced depression symptoms and 46% (*n* = 1138/2489) experienced anxiety. The proportion of respondents with depression symptoms increased slightly before the second lockdown (*n* = 1072/2342, 46% vs. *n* = 1033/2489, 41%); the proportion of patients with anxiety was stable (*n* = 1105/2342, 47% vs. *n* = 1138/2489, 46%).

During the first lockdown, 25% of respondents (*n* = 612/2489) respondents had a COVID-19 test. Of those tested, 4% (*n* = 109/2489) had a positive result. Not surprisingly, those numbers increased during the second lockdown: among the 35% (*n* = 825/2342) of tested patients, 8% (*n* = 176/2342) revealed to be positive for COVID-19.

**Figure 2:**
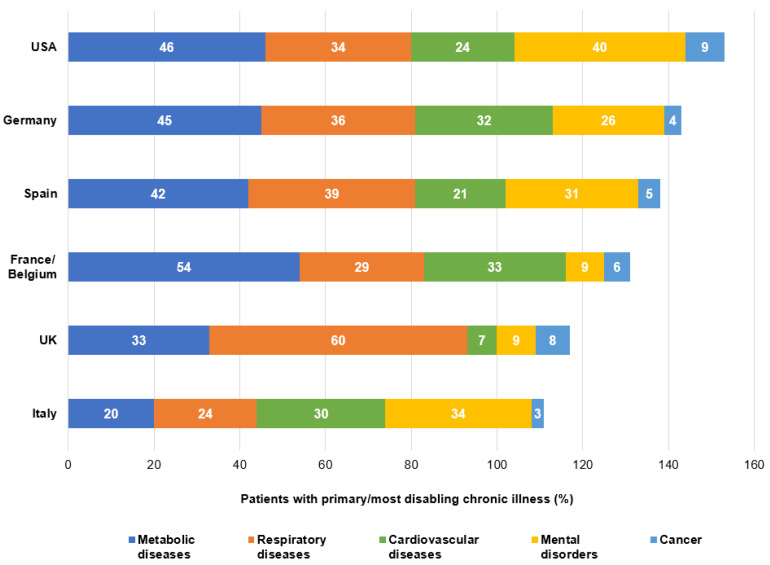
Percentage of patients with non-communicable diseases (NCDs) per country. USA, United States of America; UK, United Kingdom.

During the first lockdown, while the most reported chronic conditions concerned respiratory diseases (*n* = 277/458, 60%) in the UK, metabolic diseases were the most cited answer in France/Belgium (*n* = 335/615, 54%), in the USA (*n* = 232/502, 46%), in Germany (*n* = 110/247, 45%), and in Spain (*n* = 149/354, 42%) (Figure 2).

### 3.2. Impact of COVID-19 Pandemic on the Medical Condition of Patients with NCDs

One out of two respondents (*n* = 1242/2489, 50%) experienced a worsening of their medical condition during the lockdowns, the main reasons reported being a reduction or stoppage of physical activities (*n* = 463/2489, 19%) followed by the normal evolution of their chronic illness (*n* = 317/2489, 13%) and the fact that they did not consult their doctor (*n* = 203/2489, 8%). Moreover, 17% of respondents (*n* = 412/2489) developed a new disease during the first lockdown. Among those participants, 83% (*n* = 340/412) contacted an HCP about it: their general practitioner (for 51% of patients (*n* = 210/412)), their specialist (pneumologist, diabetologist, etc.) (*n* = 111/412, 27%), or a healthcare professional in a hospital emergency center (*n* = 75/412, 18%). Compared to the first lockdown, more specialists (151/381, 40% for wave 2 vs. *n* = 111/412, 27% for wave 1) and pharmacists (*n* = 47/381, 12% for wave 2 vs. *n* = 29/412, 7% for wave 1) were contacted by the respondents because of a new condition that had developed during the second lockdown.

### 3.3. Impact of COVID-19 Pandemic on the Behaviors of Patients with NCDs

In total, 92% (*n* = 2296/2489) of participants implemented solutions to take care of their medical condition during the first lockdown. Most of them (2057/2489, 83%) followed recommendations from health authorities, while 31% (*n* = 764/2489) of respondents scheduled a teleconsultation to avoid having to go to the hospital or the doctor’s office, and 16% (*n* = 408/2489) used an app on their smartphone, an alarm, or a pill organizer to remind them to take their medications. The percentage of patients using teleconsultation decreased to 23% (*n* = 536/2342) during the second wave. Additionally, 10% (*n* = 240/2489) of participants mentioned that an informal (non-professional) carer had helped them. Between the first and the second lockdowns, a reduction in the compliance of the health authority recommendations was observed (*n* = 2, 057/2489, 83% for wave 1 vs. *n* = 1611/2342, 69% for wave 2).

**Figure 3:**
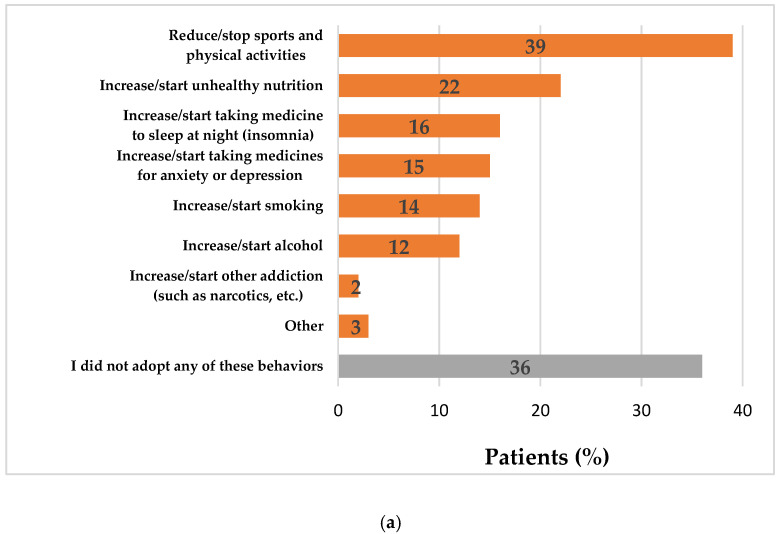

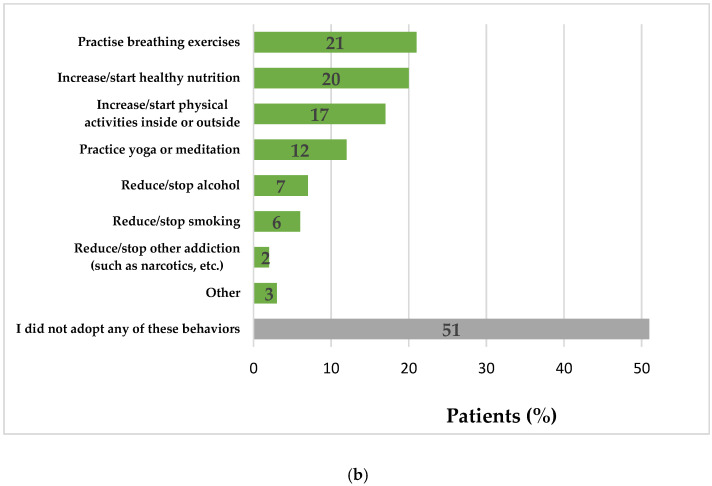
Negative (**a**) and positive (**b**) behaviors adopted during the lockdown by patients affected by a non-communicable disease (*n* = 2489, wave 1).

As shown in Figure 3a, 64% (*n* = 1601/2489) of respondents adopted a negative behavior during the lockdowns: 39% (*n* = 967/2489) of them reduced or stopped physical activities, 22% increased or started unhealthy nutrition (*n* = 539/2489), 16% increased or started taking medicine to sleep at night because of insomnia (*n* = 403/2489), 15% increased or started taking medicines for anxiety or depression (*n* = 367/2489) while some patients started or increased smoking (*n* = 352/2489, 14%) or alcohol (306/2489, 12%). On the contrary, half of respondents (*n* = 1231/2489) adopted at least one positive behavior during the lockdowns: 21% (*n* = 513/2489) practiced breathing exercises, 20% (*n* = 492/2489) increased or started healthy nutrition, 17% (*n* = 424/2489) increased or started physical activity inside or outside, and 12% (*n* = 299/2489) practiced yoga or meditation (Figure 3b). In total, 36% of patients (*n* = 891/2480) adopted simultaneously negative and positive behaviors, for instance, the 5% (*n* = 47/891) of participants who increased/started healthy nutrition and reduced/stopped sports and physical activities during the same period.

### 3.4. Impact of COVID-19 Pandemic on Mental Health of Patients with NCDs

**Figure 4:**
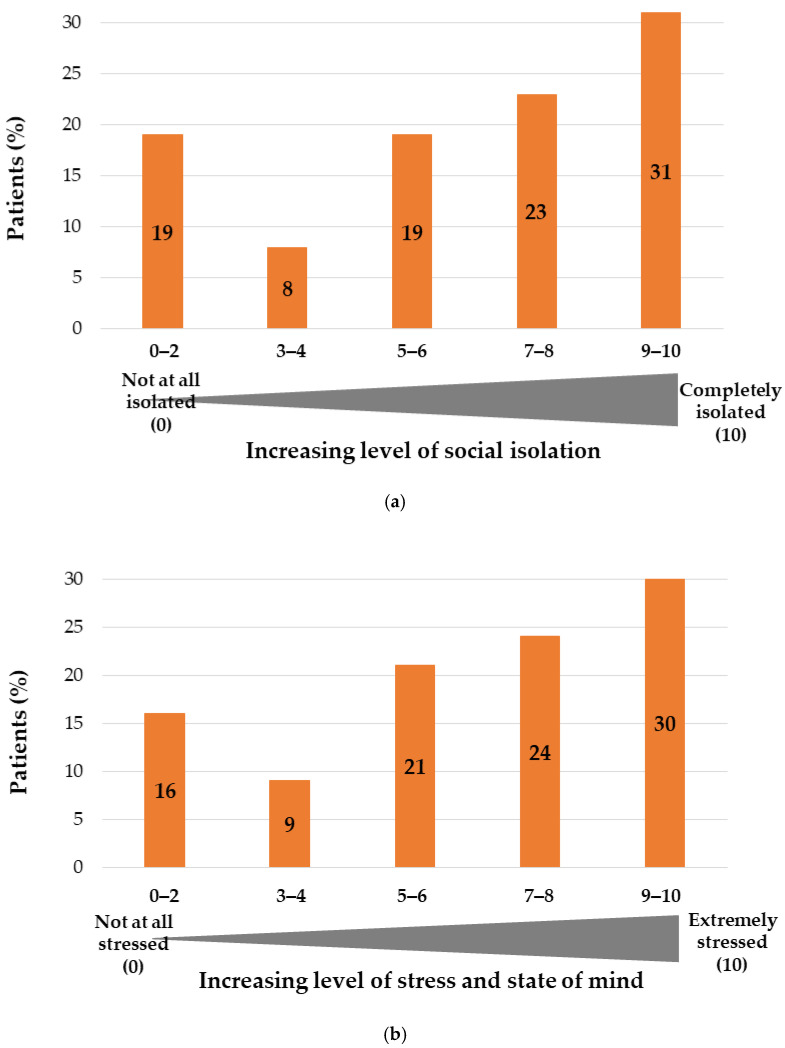
(**a**) Level of social isolation during lockdown and (**b**) impact of lockdown on stress (*n* = 2489, wave 1).

During the first lockdown, 54% (*n* = 1330/2489) of respondents mentioned that they felt very or completely socially isolated during the lockdown (answered levels between 6 and 10 on an increasing 10-step scale, with 10 = “completely isolated”) (Figure 4a); the mean level of social isolation reached 6.2 out of 10.0 (range, 0–10). In the UK and USA, respondents reported higher social isolation levels than in Europe (mean of 6.9 and 6.7/10.0 vs. 6.0/10.0, respectively) (*p*-value < 0.05).

When asked about their stress level and state of mind, 54% (*n* = 1346/2489) of respondents mentioned that both parameters had been strongly impacted by the lockdown (a rate higher or equal to 7 on a 10-step scale, with 10 = “extremely stressed”), with a mean stress level of 6.3 out of 10.0 (range: 0–10) (Figure 4b). A trend toward a higher impact of the pandemic on stress level and state of mind was observed in the USA (mean, 6.7) and in the UK (mean, 6.5) compared to Europe (mean, 6.2), although in Spain, a mean stress level and state of mind of 6.9 out of 10.0 was reported (*p*-value < 0.05).

**Figure 5:**
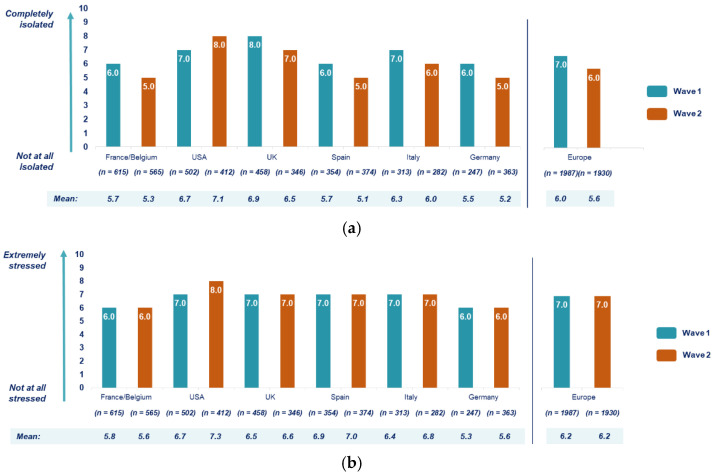
(**a**) Level of social isolation (median) during lockdown per country and wave and (**b**) impact of lockdown on stress per country and wave (*n* = 2489, wave 1; *n* = 2342, wave 2). USA, United States of America; UK, United Kingdom.

During the first lockdown, the feelings of social isolation of the respondents tended to be higher in the USA (mean, 6.7 out of 10.0) compared to European countries (mean, 6.0 out of 10.0); similar outcomes have been observed in the impact of the pandemic on the stress level and the state of mind of patients (*p*-value < 0.05) (Figure 5). This difference increased between the first and second lockdowns (Figure 5).

Among the patients who experienced depressive symptoms before the pandemic, these symptoms worsened during the lockdowns (mean, 6.2/10.0 with 10 = “Extremely worsened”; range: 0–10). Similarly, in respondents who previously had anxiety, this symptom increased during lockdown (mean, 6.5/10.0; range: 0–10). Younger patients (≤40 years) tended to experience a worsening of their depressive symptoms and anxiety compared to the older ones (>50 years) (*p*-value < 0.05). This result was observed in both waves.

In addition to depression and anxiety symptoms, 19% of respondents (*n* = 475/2489) reported having developed a mental health problem during the first lockdown and 23% (*n* = 536/2489) during the second lockdown. Patients from the USA tended to be much more affected than patients living in France/Belgium (*n* = 127/502, 25% vs. *n* = 70/615, 11% in the wave 1 and *n* = 134/412, 33% vs. *n* = 82/565, 15% in wave 2).

### 3.5. Impact of COVID-19 Pandemic on the Care Pathway of Patients with NCDs

During the first lockdown, patients with NCDs had to change the frequency of their visits to their physician: 38% (*n* = 958/2489) of respondents said they were seeing their physician less than usual, whereas 16% (*n* = 393/2489) reported having consulted them more than usual. Compared to the first lockdown, the proportion of patients who consulted their physician less than usual decreased from 38% (*n* = 958/2489) to 23% (*n* = 546/2342) during the second lockdown.

Overall, almost 30% of respondents (*n* = 749/2489) declared having had difficulties in finding an available physician during the first lockdown. Moreover, medical visits or procedures were strongly impacted, as 52% of respondents (*n* = 1298/2489) reported that long-planned medical consultations or surgeries had been canceled or rescheduled since the start of the pandemic. However, between both waves of the study, the proportion of cancelled or postponed medical consultations or procedures decreased from 52% (*n* = 1298/2489) to 32% (*n* = 740/2342).

**Figure 6:**
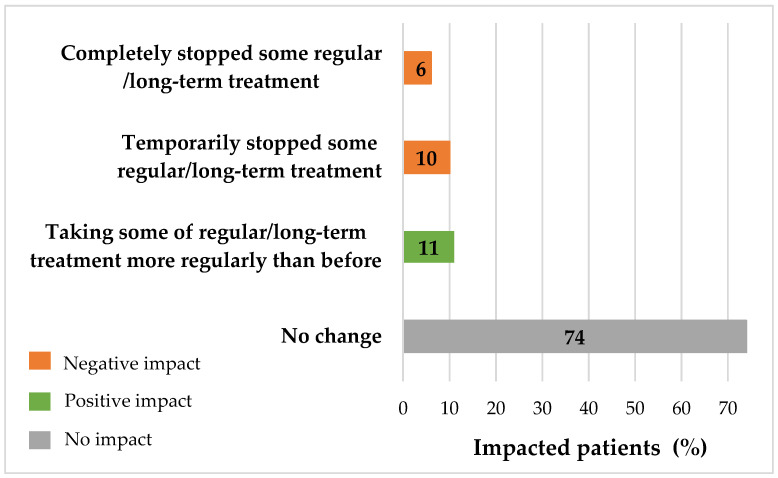
Impact of lockdown on treatment intake (*n* = 2489, wave 1).

During the first lockdown, 26% (*n* = 572/2242) of respondents under a regular/long-term treatment reported an impact of the pandemic on their regular/long-term treatment intake: 6% (*n* = 136/2242) completely stopped some of their treatments and 10% temporarily stopped some of them (*n* = 230/2242), while 11% (*n* = 242/2242) took them more regularly than they usually did (Figure 6). Additionally, 18% of respondents (*n* = 407/2242) reported having difficulties in finding their treatment at the pharmacy during the lockdown.

### 3.6. Access and Need for Disease-Specific Information

**Figure 7:**
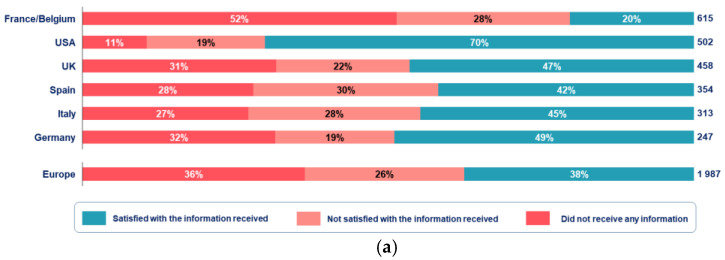

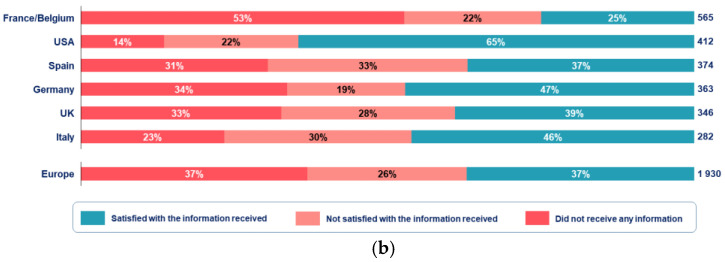
Satisfaction towards information related to condition during the first wave (**a**) and during the second wave (**b**) (*n* = 2489, wave 1; *n* = 2342, wave 2). USA, United States of America; UK, United Kingdom.

In all, 74% of patients had concerns about the pandemic, with 21% (*n* = 503/2489) of them feeling frustrated most of the time. Considering the access to specific information related to their chronic illness in the context of the pandemic, 31% of patients (*n* = 776/2489) did not receive any illness information, and 25% of them (*n* = 614/2489) reported receiving insufficient information. Of those participants, 59% (*n* = 822/1390) wished they had received additional information regarding the risks associated to their medical condition, while 32% (*n* = 445/1390) requested advice on how to deal with the end of the lockdown, and 29% (*n* = 408/1390) needed more information on available psychological support. During both waves, patients living in the USA tended to be more satisfied regarding the access to specific information related to their primary chronic illness compared to patients from Europe (Figure 7).

Beyond the need for more reliable and complete information from health authorities (*n* = 1192/2489, 48% of respondents) or in the media (*n* = 794/2489, 32%), respondents also expressed their needs in terms of solutions and services to better cope with the COVID-19 pandemic. Patients wished to have had unrestricted access to their family or close friends during the lockdown (*n* = 660/2489, 27%), access to monthly consultations with their specialists (*n* = 587/2489, 24%), or easier access to telemedicine (*n* = 579/2489, 23%).

**Figure 8:**
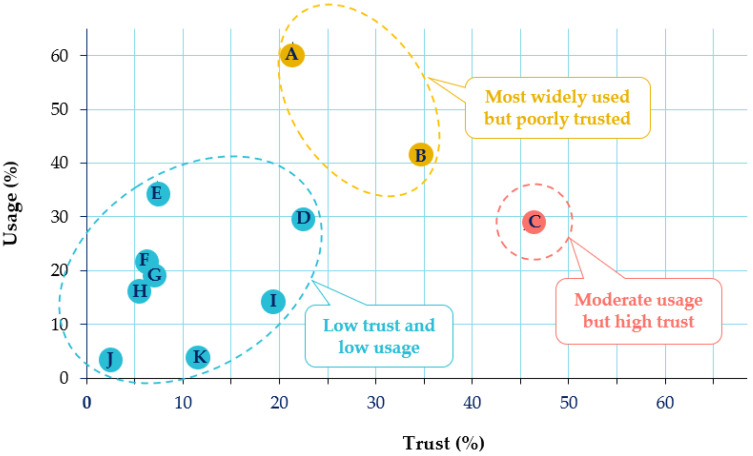
Sources of information about the COVID-19 pandemic and trust level of patients affected by a non-communicable disease in these sources. Yellow cluster: (A) television; (B) government/health authority sites. Red cluster: (C) physicians. Blue cluster: (D) health websites; (E) social networks; (F) general websites; (G) press; (H) radio; (I) other health professionals; (J) others; (K) none. (*n* = 2489, wave 1).

As shown on Figure 8, though television was used by 61% of patients (*n* = 1512/2489) as a source of information, it was poorly trusted (only by 21% of respondents, *n* = 532/2489). On the contrary, physicians were the most trusted source of information (*n* = 1153/2489, 46%) but could be used more often as they were as the fifth most used source by the patients. This outcome did not significantly change between both lockdown situations.

### 3.7. Impact of the Pandemic and Patients’ Need Depending on Patients’ Profile

To identify the groups of patients with similar patterns of responses to the questions, a clustering analysis on the categorical data was performed through a multivariate analysis. Based on the answers collected through the questionnaire, three different clusters emerged for each wave: younger highly impacted patients, middle-aged moderately impacted patients, and less impacted older patients (Table 3) (*p*-value < 0.05). In comparison to Cluster 3 (older patients), respondents in Clusters 1 and 2 suffered more depressive and anxiety symptoms before lockdown and had self-reported disabling medical conditions, felt isolated and stressed, had a worsening of their medical condition, had seen their physician more than usual, were very affected by the cancellation of their consultations or surgeries, and developed a new disease (including a new mental illness) more frequently) (*p*-value < 0.05). Moreover, during the first lockdown, respondents in Cluster 2 were those who were the least satisfied with the access to specific information related to their chronic pathology (63% unsatisfaction vs. 49% in Cluster 1 and 55% in Cluster 3). During the second lockdown, no major differences were observed in these three clusters, except that patients from Cluster 2 reported mental diseases as primary chronic illness instead of respiratory diseases and cancers.

## 4. Discussion

This online patient survey conducted via the Carenity online communities among adults self-reporting as having an NCD showed how the disruptions in health services due to the pandemic strongly affected the daily life of this exposed population. Statistical methods involving clustering analysis, social desirability, and influence given the general increased awareness of people with existing NCDs being more at risk during the pandemic. With the rapid spread of COVID-19 across the world, patients followed health authority recommendations and adapted their behaviors to the new situation (lockdown). A majority of the patients adopted negative behaviors such as reducing or stopping physical activities, starting or increasing unhealthy nutrition, and increasing smoking or alcohol use. Similar negative behaviors have been already reported in numerous European countries [21,22,23,24,25]. Compared to an active lifestyle, sedentary living and its associated comorbidities—for instance, obesity—have been linked to an increased risk of NCD development [3].

Social isolation was mostly enforced through governmental policies on social distancing; in our study, social isolation affected a lot of patients (54%) living with NCDs, especially in the UK and USA, and many patients wished to have unrestricted access to their family and close friends during lockdown. It should be noted, though that if social isolation had been largely overlooked in general, specific initiatives were developed to address this issue, for instance, the UK Campaign to End Loneliness [26]. Nevertheless, social isolation might have also been self-imposed by NCD patients because of the fear of contagion in healthcare settings—this population was described as more vulnerable to COVID-19 infection in comparison to the overall population [27]—as well as an initial period of uncertainty without an understanding of COVID-19 infection [10,11]. Increased feelings of loneliness were described, particularly in older individuals who were at a higher risk for developing mental health disorders [28]. Social isolation might have additionally increased the stress status of patients living with NCDs, as 54% of respondents reported an increased stress level during the lockdowns. Consequently, during the lockdowns, depressive symptoms and anxiety increased 51% and 56%, respectively, in respondents living with NCDs, especially in the younger population. In comparison, in a large literature review including 16 studies and covering the first seven months of the pandemic, Lakhan et al. described the prevalence of depression (20%), anxiety (35%), and stress level (53%) in the general population [29]. A Brazilian study described that patients with depression were at a higher risk for the incidence of unhealthy diet behavior during lockdown [30]. In the pandemic context, pre-existing mental disorders worsened among respondents, and additional new mental disorders appeared. Similar outcomes in terms of increased NCD risk factors and worsened clinical symptoms have been also described by Palmer et al. [28]. Our study raises the question of the long-term consequences of medical conditions worsening, such as the suicide arising in this endangered population.

Additionally, the COVID-19 pandemic had a strong impact on patients’ access to HCPs and their treatments. Most medical practices as well as health care and rehabilitation services suddenly closed or scaled down non-urgent visits while hospitals were overwhelmed with severe COVID-19 cases, lacking staff, PPE, and essential medicines. Additionally, confinement restrictions strongly limited the access of NCD patients to health care and medication, which increased their uncertainty. Especially in the early days of the lockdown, this situation, which led NCD patients unattended, forced them to change their access to care. In our study, respondents reported seeing their physician less than usual, having difficulties finding an available physician, and trouble finding their treatment at the pharmacy during the first lockdown. Subsequently, patients often modified the intake frequency of their regular or long-term treatment, which might have decreased their treatment compliance and increased the risk of iatrogenesis. This was also shown in other surveys [31]. Limited access to their HCP jeopardized patient adherence to treatment, potentially resulting in the long-term worsening of their chronic condition and contributing to the high burden of NCDs [23]. The annual costs of medication non-adherence have been estimated to be as high as EUR 1.25 billion in Europe and USD 290 billion in the USA [32]. Additionally, many patients had to cope with the fact that many medical surgeries and consultations were postponed or canceled (52% during the first lockdown in our study).

Soon after the start of the pandemic, triaging was the most widely adopted strategy worldwide to ensure high risk NCD patients continuing access to critical monitoring services and treatments without increasing the risk of COVID-19 contagion at hospitals [33]. For this purpose, the COVID-19 patient management algorithm published by the WHO might be applicable to NCD patients [34]. Months after the start of the pandemic, the frequency of physician consultations was still reduced, and patients had to adapt their consultation mode to get appropriate information on their chronic diseases. Therefore, more patients started using telemedicine (advice by telephone or online means) for their medical visits [35,36,37,38]. Telemedicine might be an additional and substitutive way of providing continuing essential healthcare services. However, as the use of telemedicine is highly dependent on the availability of technology and expertise, this technology might not be accessible to the elderly population; guidance and support are therefore urgently needed [10]. Health specialists and pharmacists might also be involved in the development of the telemedicine especially [39]; due to the pandemic in the past year, the rapid growth of telemedicine has renewed the debate of physician licensing in many countries [40]. In that respect, this pandemic might have been an opportunity to learn how to leverage healthcare technologies [28].

To better cope with their chronic diseases during the pandemic, patients needed more specific information, especially on how COVID-19 infection might impact their current pathology in terms of the associated risks and appropriate care management during lockdown. At the beginning of the pandemic especially, patients had difficulties distinguishing reliable medical facts and misinformation among the huge amount of available information. During the pandemic, governmental agencies such as the WHO were providing relevant information on patients with NCDs and the COVID-19 pandemic [10,11]. In our study, while television was the most used source of information during the pandemic, it was not very well trusted; on the other hand, if physicians were the most trusted source of information by patients on their chronic disease, they were not consulted enough. In addition to physicians, different sources with validated information, such as the pharmacist or patient advocacy groups, related to specific pathologies were available during the lockdowns.

As rehabilitation care services as well as health and social care at home were the most commonly disrupted services worldwide [12], NCD patients were essentially relying on non-professional carers/caregivers—most of them being family members—for daily care, and in some cases, including technical medical care as well as for keeping physicians updated on the patient’s condition. Due to the pandemic, the disruption of care services dangerously aggravated a pre-existing situation and led to an overreliance on carers for long-term care, jeopardizing their situation [41]. Considering the number of people living in the same household as the respondents and the deterioration reported by the latter concerning their health and well-being, family members living under the same roof were more likely to be impacted by this acute situation through additional stress and informal care provisions. The lack of awareness of the role of the informal carer/caregiver—often taken for granted by the carer as well as by the person in need of care—was most likely to explain the fact that only 10% of respondents mentioned explicitly having asked an informal carer for help. Most informal carers/caregivers had to cancel or reschedule their own medical consultations or surgeries to manage the health care of their close relatives with NCDs during the pandemic and reported having experienced more problems in terms of reconciling work and care since the start of the coronavirus pandemic [42,43,44]; this impact of the pandemic on informal carers/caregivers—on top of their daily burden in non-pandemic times [45]—has been largely overlooked. Both patients and informal carers/caregivers suddenly had increased feelings of isolation and were in urgent need for information to educate themselves on rehabilitation procedures. During the pandemic in France, caregiver associations developed free-accessible online dedicated videos and print documents to train chronic patients and their non-professional/informal carers/caregivers on how to manage their essential care and condition in different life situations [46]. Emerging situations like the COVID-19 pandemic especially revealed that such educational programs are crucial for patients and informal carers to lower their anxiety and stress level, therefore reducing the risk of comorbidity development. In an assessment of the situation performed by the WHO in patients living with NCDs, several countries worldwide requested explicit support in developing communication materials addressing NCDs and their risk factors in the context of the pandemic [10].

This pandemic therefore stressed the long-needed support of informal carers/caregivers and the need for integrated care supported by the WHO and the European healthcare system [47,48]. Patients with NCDs, especially those presenting with severe conditions and/or multimorbidities, rely on integrated care programs; those were greatly affected during the pandemic as healthcare services were very busy managing the COVID-19 crisis. This might have had devastating consequences, particularly among NCD patients requiring regular symptom monitoring and the adjustment of complex drug regimens [47]. Although the care management of patients living with NCDs is mainly a patient-centered approach, integrated care including all of the relevant primary to tertiary health services is a key factor, especially for patients with more severe pathologies and multimorbidities, who are more disabled. In fact, patients with NCDs might be described as living in an ecosystem, in which central informal carers/caregivers play a key role, and therefore, are dependent on all of the surrounding health care management services; the coordination of care among the different health care partners facilitates the appropriate and timely delivery of health care services to NCD patients [49]. Integrated care means HCPs, secondary/hospital care specialists, pharmacists, rehabilitation specialists, residential care specialists, mental health specialists, informal carers/caregivers as well as patient and caregiver associations work in collaboration to improve care management for patients with NCDs. This point raises the need for a health care system reform to implement or strengthen NCD integrated care management programs, especially in terms of linking hospital and private practices or implementing care managers, such as in the UK, for the organization of a patient’s daily life after hospital discharge. Governments around the world need to understand how to support health services, align on clear and concise healthcare messaging from a globally established reliable source of information, and have emergency plans in place to mitigate the impact of any future sanitary crises [10,50,51].

Differences in the health care systems could also explain the differences observed. Health coverage in Europe is universal. Having different structures of interactions between insurers, providers, and patients, all European healthcare systems aim to provide care to everyone on the grounds of free access, equality and equity, and fairness: no matter how much patients earn, patients receive a basic package. Unlike Europe, not all American citizens have access to publicly-funded insurance; government funds are available for certain Native American tribes, military families, and veterans. There is also national health insurance, called Medicare, which covers people over 65 and some people with disability status, people with end-stage renal disease, and amyotrophic lateral sclerosis [52]. These differences could especially explain the impact in the access to HCPs or on the social isolation/anxiety that have been described more often in the US patients. Additionally, disparities in terms of satisfaction towards the information received could be mostly due to the differences in the health care systems and reimbursement access between Europe and the USA.

This study presents several limitations, mostly inherent to its design. The following bias should be mentioned: selection bias (underrepresentation of older patients on Carenity communities or patients without online access or high-IT and literacy bias, overrepresentation of patients worrying about their health status, and members of Carenity communities), self-reported perceptions and symptoms, and recall bias, especially concerning the retrospective aspect of the study (wave 1). However, as the answering of the questionnaire was done directly by the patients in absence of their physician, the desirability bias was greatly limited. Moreover, the high similarity of the results obtained in both waves validates our study concept. Finally, belonging to a patient community likely reflects the need to self-assess and compare the levels of the disease. Poorly controlled, highly symptomatic, and heavily treated patients may then be overrepresented, and our findings should be extrapolated only cautiously. However, the majority of people use the Internet. In 2019, it was estimated that 86.7 percent of people living in developed countries used the internet [53]. Moreover, it has been shown that Carenity communities, when compared with the SNIIRAM database (a quantified claim database available in France where all individual refunding acts are listed but without any disease-related information), reflect the main characteristics of online users willing to share their experience with a disease, with an over-representation of female patients aged from 25 to 54 [54]. The study population was based on patients registered on the Carenity platform from Europe and USA and did not include other relevant sampling procedures and methods. Despite the study recruitment comprising of a homogeneous population of patients through the Carenity platform, no generalizations of the study can be assumed.

## 5. Conclusions

During the pandemic, healthcare services disruptions coupled with newly adopted negative behaviors put patients living with NCDs at high risk for the worsening of their chronic condition or developing multimorbidities. The burden of NCDs, which increased during the pandemic, has recurrent health and economic consequences for the patients’ ecosystems. Hopefully, lessons from the COVID-19 pandemic will be learned, and emergency plans will be put in place to protect the NCD vulnerable population against upcoming outbreaks. NCDs were included for the first time in the United Nations 2030 agenda for sustainable development as a health priority to reinforce WHO engagement by 2030; the aim is to reduce premature mortality from NCDs by one third through prevention and treatment as well as to promote mental health and well-being [55]. Our study highlights the urgent need to restore a patient centric approach to health care—which has been clearly endangered by the COVID-19 pandemic—and to empower patients affected by non-communicable diseases (NCDs).

## Figures and Tables

**Table 1:** Sociodemographic profile *.

Variables	First Wave (*n* = 2489)	Second Wave (*n* = 2342)
Gender, *n* (%)		
Female	1602 (64)	1396 (60)
Male	887 (36)	946 (40)
Mean age, years (range)	53 (18–91)	52 (18–90)
Age groups, years		
≤40	528 (21)	560 (24)
41–50	423 (17)	393 (17)
51–60	698 (28)	652 (28)
61–70	612 (25)	531 (23)
>70	228 (9)	206 (9)
Country of residence, *n* (%)		
Belgium	4 (<1)	14 (<1)
France	611 (25)	551 (24)
Germany	247 (10)	363 (15)
Italy	313 (13)	282 (12)
Spain	354 (14)	374 (16)
United Kingdom	458 (18)	346 (15)
United States of America	502 (20)	412 (18)
City size, *n* (%)		
Metropolitan city ^a^	542 (22)	533 (23)
Large city ^b^	333 (13)	285 (12)
Mid-sized city ^c^	568 (23)	580 (25)
Small city ^d^	637 (26)	568 (24)
Rural town ^e^	357 (14)	339 (14)
Other	52 (2)	37 (2)
Housing type, *n* (%)		
House with a yard	1418 (56)	1260 (54)
Apartment	814 (33)	831 (35)
House without a yard	125 (5)	111 (5)
Studio apartment	63 (3)	90 (4)
Other	69 (3)	50 (2)
Number of people per household ^f^,		
Mean number (range)	2.7 (1–10)	2.6 (1–9)
*n* (%)		
1	469 (19)	451 (19)
2	944 (38)	849 (36)
3	478 (19)	466 (20)
>3	598 (24)	576 (25)
Number of children per household		
Mean number (range)	0.5 (0–4)	0.5 (0–5)
*n* (%)		
0	1754 (71)	1599 (68)
1	373 (15)	378 (16)
2	251 (10)	257 (11)
≥3	79 (3)	79 (3)

* Percentages may not equal 100 because of rounding; ^a^ >1,000,000 inhabitants; ^b^ 100,000 to 1,000,000 inhabitants; ^c^ 20,000 to 100,000 inhabitants; ^d^ 2000 to 20,000 inhabitants; ^e^ <2000 inhabitants; ^f^ Including children.

**Table 2:** Clinical characteristics *.

Variables ^a^, *n* (%)	First Wave (*n* = 2489)	Second Wave (*n* = 2342)
Chronic diseases		
Metabolic diseases	1042 (42)	989 (42)
Respiratory diseases	930 (37)	772 (33)
Cardiovascular diseases	603 (24)	691 (30)
Mental disorders	577 (23)	597 (25)
Cancer	155 (6)	170 (7)
Most frequent chronic conditions		
Type 2 diabetes	832 (33)	693 (30)
Arterial hypertension	575 (23)	419 (18)
Asthma	562 (23)	543 (23)
COPD ^b^	459 (18)	312 (13)
Depression	412 (17)	301 (13)
Disability level of disease ^c^, mean (range)	5.5 (0–10)	5.5 (0–10)
0–1	366 (15)	336 (14)
2–3	370 (15)	360 (15)
4–5	621 (25)	560 (24)
6–7	620 (25)	603 (26)
8–10	512 (20)	483 (21)
Intake of regular/long-term treatments		
Yes	2242 (90)	2114 (90)
No	247 (10)	228 (10)
Depressive symptoms before lockdown		
Yes	1033 (41)	1072 (46)
No	1456 (59)	1270 (54)
Anxiety before lockdown		
Yes	1138 (46)	1105 (47)
No	1351 (54)	1237 (53)
COVID-19 testing		
Yes	612 (25)	825 (35)
No	1877 (75)	1517 (65)
COVID-19 testing results		
Positive	100 (16)	166 (20)
Negative	503 (82)	649 (79)
Positive and negative ^d^	9 (2)	10 (1)

* Percentages may not equal 100 because of rounding; ^a^ Except other indicated; ^b^ Chronic obstructive pulmonary disease; ^c^ Ranking from 0 (not disabling at all) to 10 (very disabling); ^d^ during the period of the survey, patients underwent several tests. some of them were positive, and others were negative.

**Table 3:** Multivariate clustering analysis. HCP, health care professional. (*n* = 2489, wave 1).

Variables	Cluster 1 (*n* = 802)	Cluster 2 (*n* = 887)	Cluster 3 (*n* = 800)
Type of Patients	Younger highly Impacted Patients	Middle-aged Moderately Impacted Patients	Less impacted Older Patients
Age (mean)	≤45 years old (40.5)	46 to 60 years old (57.5)	>60 years old (60.1)
Gender	59% of women	79% of women	54% of women
Number of chronic diseases	less than 3	more than 3	less than 3
Primary chronic diseases	mental diseases	respiratory diseases and cancers	metabolic and cardiovascular diseases
Living in…	Italy, Spain and the USA	the UK and France/Belgium	France/Belgium and Germany
Depressive and anxiety Symptoms before lockdown	yes	yes	no
More likely to live in big or small cities	big	small	small
Disabling medical condition	moderately disabling	very disabling	not very disabling
Adoption of bad habits during lockdown	yes	yes	no
Level of isolation and stress	isolated and stressed	isolated and stressed	not isolated and not stressed
Worsening of the medical condition	yes	yes	no
Have seen their physician…	more thanusual	more than usual	As usual
Difficulty in finding an available HCP	yes	no	no
Affected by cancellation of consultations or surgeries	very affected	very affected	less affected
Development of a new illness	yes	yes	no
Development of a new mental illness	yes	yes	no

## Data Availability

The data presented in this study are available on request from the corresponding author. The data are not publicly available due to privacy laws.
